# Evaluation of salivary oxidative parameters in overweight and obese young adults

**DOI:** 10.1590/2359-3997000000227

**Published:** 2016-11-07

**Authors:** Eduardo Ottobelli Chielle, Jeferson Noslen Casarin

**Affiliations:** 1 Laboratório de Bioquímica Clínica Universidade do Oeste de Santa Catarina São Miguel do Oeste SC Brasil Departamento de Ciências da Saúde, Laboratório de Bioquímica Clínica, Universidade do Oeste de Santa Catarina (Unoesc), São Miguel do Oeste, SC, Brasil

**Keywords:** Obesity, biomarkers, oxidative stress, saliva, antioxidants

## Abstract

**Background:**

Obesity is characterized by a deposition of abnormal or excessive fat in adipose tissue, and is linked with a risk of damage to several metabolic and pathological processes associated with oxidative stress. To date, salivary oxidative biomarkers have been minimally explored in obese individuals. Thus, the aim of this study was to assess the concentrations of salivary oxidative biomarkers (ferric-reducing antioxidant power, uric acid, sulfhydryl groups) and lipid peroxidation in obese and overweight young subjects.

**Materials and methods:**

Levels of lipid peroxidation, ferric-reducing antioxidant power, uric acid, and SH groups were determined in the saliva and serum of 149 young adults, including 54 normal weight, 27 overweight, and 68 obese individuals. Anthropometric measurements were also evaluated.

**Results:**

Salivary levels of ferric-reducing antioxidant power, sulfhydryl groups, and lipid peroxidation, as well as serum levels of ferric-reducing antioxidant power, uric acid, and lipid peroxidation were higher in obese patients when compared with individuals with normal weight. There were correlations between salivary and serum ferric-reducing antioxidant power and salivary and serum uric acid in the obese and normal-weight groups.

**Conclusions:**

Our results indicate that the increase in salivary levels of ferric-reducing antioxidant power, sulfhydryl groups, and lipid peroxidation, and serum levels of ferric-reducing antioxidant power, uric acid, and lipid peroxidation could be related to the regulation of various processes in the adipose tissue. These findings may hold promise in identifying new oxidative markers to assist in diagnosing and monitoring overweight and obese patients.

## INTRODUCTION

Reactive oxygen species (ROS) and reactive nitrogen species (RNS) play an important role in cell signaling and metabolic pathways in physiological conditions. On the other hand, decreased antioxidant levels and/or increased production of reactive metabolites may disrupt homeostatic processes and lead to oxidative damage (1,2). Oxidative/nitrosative stress may have a serious impact on cell viability and induce cellular responses leading to cell death (3). Many studies have shown a connection between oxidative molecular damage and pathophysiological mechanisms associated with severe diseases such as atherosclerosis (4), neurodegenerative disorders (5), and diabetes, and an important role in the etiopathogenesis of inflammatory diseases (6). There is also a relationship between oxidative stress and aging processes (7).

Obesity is associated with an increase in oxidative stress, defined as an increased load of free radicals comprised of ROS and RNS generated during cellular metabolism (8). These free radicals are chemically reactive molecules that may damage cellular proteins, membranes, and DNA. The increase in oxidative stress is considered to be involved in the pathogenesis of insulin resistance and type 2 diabetes (T2DM) associated with obesity (9,10). Data from studies with cell culture systems have shown that products of oxidative stress impair insulin-mediated translocation of GLUT4 in myotubes and adipocytes and suppress the transcription of the insulin gene in β cells and adiponectin in adipocytes (11,12).

On the other hand, most cells have an adequate protective system to circumvent harmful oxidative events. This system is composed of antioxidant enzymes (superoxide dismutase, catalase, and many other peroxidases) and nonenzymatic antioxidants, including the glutathione (SH groups), uric acid, ascorbate, and ferric-reducing antioxidant power (FRAP) (13). Was observed a progressive increase TBARS levels in accordance with the increase in body weight and progressive decrease in SH groups and FRAP according to body weight gain (14). The increased oxidative stress in vascular walls is involved in the pathogenesis of atherosclerosis, hypertension and induces damage to cell structures, including membranes, proteins, and ADN and this contributes to disorders cardiometabolic (15).

The major antioxidant capacity toward named oxidants is represented by numerous thiol groups (SH groups) in intracellular and extracellular compartments. At the same time, thiols are also the major targets for ROS and RNS. It is well known that the serum SH groups levels are modified in many diseases, including obesity, indicating that SH groups content is a useful biochemical marker of “in vivo” oxido-reduction reactions (16). Membrane SH groups are liable to be modified by different oxidants or alkylating agents, increasing their membrane permeability to different ions, such as Ca^++^, which can promote excitotoxicity, by RNS and ROS that lead to lipid peroxidation, increasing the levels of TBARS and decreased concentration of SH groups in membrane proteins (17). Thiol-containing molecules with SH bonds such as glutathione (GSH) suppress oxidative damage and involve the maintenance of the cell redox status. In healthy tissues, these antioxidants work in cooperative to maintain the pro oxidant–antioxidant balance and prevent tissue damage and disease (18).

The saliva also has antioxidant properties attributed to its composition of enzymatic (mostly peroxidase system) and nonenzymatic compounds (uric acid, glutathione, sialic acid), which may be determined in salivary samples (19,20). Sampling of human saliva is an attractive means to diagnose and monitor diseases since the saliva may be collected easily and noninvasively. Analysis of the saliva may also be beneficial to predict the progression of future diseases (14,21,22). As a clinical tool, saliva has many advantages over serum, including ease of collection, storing, and shipping. Saliva is composed of organic and inorganic elements and represents an important fluid in the oral cavity that has been used as a sample to diagnose and control the treatment of systemic diseases and disorders (23). Measurement of oxidative biomarkers in the saliva to examine numerous clinical conditions has increased over the last decade (24).

Thus, considering the fact that obesity is a chronic state that involves diverse pathways such as oxidative stress, inflammation, and endothelial dysfunction, the aim of this study was to assess the salivary and serum levels of oxidative biomarkers in obese and overweight subjects and analyze the correlation between salivary and serum parameters.

## MATERIALS AND METHODS

### Study population

Participants were recruited from January to August 2014 in São Miguel do Oeste located in south of Brazil. The protocol of the study was approved by the Ethics Committee of the University of West Santa Catarina (UNOESC, Nº 219.091) and all participants provided writ- ten informed consent. Experiments were performed in 149 subjects. A total of 54 normal weight subjects with gender-matched healthy volunteers served as a control group (32 females and 22 males). The subjects with increased weight were divided in two subgroups, matching for sex, age, and body mass index, and were enrolled as follows: 1) 27 overweight subjects (17 females and 10 males); 2) 68 obese young subjects (41 females and 27 males). The participants were non-smokers and were not using any medications, as shown in Table 1.

**Table 1:** Baseline characteristics of study participants

		Groups	

	Normal weight	Overweight	Obese
N	54	27	68
Male/Female	22/32	10/17	27/41
Age (years)	21.0 (19.8-24.0)	24.0 (21.0-26.0)	25.0 (22.0-27.0)
Weight (kg)	60.1 ± 9.4	77.2 ± 7.0^a^	97.7 ± 16.0^ab^
Height (cm)	167.8 ± 7.3	167.0 ± 8.3	166.8 ± 10.5
BMI (kg/m^2^)	20.9 (19.3 – 22.6)	28.1 (26.5 – 28.7)^a^	34.1 (32.4 – 37.5)^ab^
NC (cm)	36.0 ± 4.3	36.0 ± 3.3	38.8 ± 3.5^ab^
WC (cm)	72.3 ± 6.8	87.7 ± 6.3^a^	104.2 ± 13.7^ab^
HC (cm)	95.7 ± 6.2	107.1 ± 5.5^a^	117.8 ± 8.9^ab^
SBP (mmHg)	120.9 ± 11.7	126.3 ± 11.2	136.7 ± 14.2^ab^
DBP (mmHg)	72.0 (67.8 – 80.0)	81.0 (73.0 – 89.0)^a^	86.0 (77.0 – 93.0)^a^
Body fat (%)	25.3 (18.9 – 28.9)	33.3 (27.4 – 36.8)^a^	38.7 (34.8 – 41.6)^ab^
Fat body mass (kg)	14.9 (12.7 – 17.9)	24.2 (21.0 – 28.3)^a^	36.1 (31.1 – 40.7)^ab^

Data are expressed as means ± SD or median (interquartile ranges). Data were processed for analysis for One-way ANOVA followed by Tukey’s test or Kruskal Wallis test followed by Dunn’s Multiple Comparison Test. BMI: body mass index; NC: neck circumference; WC: waist circumference; HC: hip circumference; SBP: systolic blood pressure; DBP: diastolic blood pressure.

^a^* p *< 0.0001 compared to normal weight group.

^b ^* p *< 0.0001 compared to overweight group.

### Anthropometric measurements

All measures were taken in the Anthropometry Laboratory at the University of West Santa Catarina, São Miguel do Oeste, SC (Table 1). Standing height (H, cm) was measured to the nearest 0.1 cm using a wall-mounted stadiometer (Charder model HM-210D). Weight (W, kg) was measured to the nearest 0.1 kg using a calibrated electronic scale (Toledo model 2124). BMI was calculated as W/H^2^ (kg/m^2^). Waist circumference (WC), neck circumference (NC) and hip circumference (HC) was measured in centimeters with a flexible tape to the nearest 0.1 cm. For AC the tape was applied above the iliac crest with the subject standing with the abdomen relaxed, arms at sides and feet together. NC for the participant remained in the same position and tape was placed on half of the neck on the hyoid bone. The percentages of fat and fat weight were determined by bioimpedance (Biodynamics Model 450). Systolic and diastolic pressure (SBP, DBP) was measured in the individual after being seated and resting for 10 minutes, with a digital apparatus and were expressed in mmHg. All measurements were taken on the left side of the body, according to standardized procedures by Weiner and Lourie (11). During the anthropometric measurements, all participants were barefoot and clothed appropriately.

### Indices and classifications

According to the World Health Organization, underweight was defined as BMI < 18.5 kg/m^2^, normal weight as BMI 18.5–24.9 kg/m^2^, overweight as BMI 25–29.9 kg/m^2^, and obesity as a BMI > 30 kg/m^2^ (12), all without comorbidities. According to Gallagher and cols. (13) % fat ≥ 20% (males) and % fat ≥ 33% (females) are the cut-points adopted to define over fatness, corresponding to overweight classification using BMI in a population of young adults. According to the National Institute for Health and Clinical Excellence guidelines, WC ≥ 102 cm for men and ≥ 88 cm for women are prerequisite risk factors for the diagnosis of the metabolic syndrome, as Waist-Hip Ratio (WHR) ≥ 0.9 for males and ≥ 0.8 females (25).

### Saliva sampling

A well defined and standardized protocol was used for collection, storage, and processing of all the samples under the exactly same conditions. Unstimulated saliva samples were collected in the morning after fasting for at least 8 h. Brushing teeth, smoking, eating or drinking anything but water for at least 60 min prior to sampling were prohibited. For the collection of salivary samples, patients were asked to put and keep the cotton swab under tongue for Laboratory analyzes 3 min and then to place it back directly into the plastic container according to the manufacture’s instruction (Salivette tubes, Sarstedt, Nümbrecht, Germany). The collections were made in the laboratory under the guidance and supervision of researchers. Immediately after collecting the saliva samples were centrifuged for 10 min at 1100g, fractionated in eppendorf and were stored at –20ºC until analysis (19). Approximately 200 µL of thawed saliva samples was processed for enzymatic and biochemical tests. To avoid the effects of protein degradation, the samples that had been thawed were not reused.

### Laboratory methods

Determination of Ferric-Reducing Antioxidant Power (FRAP): salivary and serun FRAP levels were measured according to Singh and cols., (20). Two hundred μL of prewarmed 37ºC FRAP reagent (1 volume of 3 mol/L acetate buffer, pH 3.6 + 1 vol of 10 mmol/L 2,4,6-tripyridyl-S-triazine in 40 mmol/L HCl + 1 vol of 20 mmol/L FeCl_3_) was mixed with 20 μL of saliva. Absorbance was read at 593 nm. Ferrous sulphate was used as standard and the concentration of FRAP was expressed in μmol/L. All other chemicals were obtained from Sigma Chemical Co. (St. Louis, MO, USA).

Determination of Thiobarbituric Acid Reactive Substances (TBARS): lipid peroxidation was estimated in plasma and salivary by measurement of thiobarbituric acid reactive substances (TBARS) according to the method of Lapenna and cols. (23), using 1% phosphoric acid and 0.6% thiobarbituric acid (TBA). The reaction product was measured spectrophotometrically at 532 nm and the results were expressed in nmol TBARS/mL.

Determination of Protein thiol groups (SH groups): protein thiol groups were assayed in salivary and plasma by the method of Boyne and Ellman (24) which consist of the reduction of 5.5’-dithio(bis-nithrobenzoic) acid (DTNB) in pH 7.0, measured at 412 nm. The results were expressed in nmol P-SH/mL.

### Statistical analysis

The data were analyzed using Statistica 6.0 software (StatSoft, Tulsa, OK, USA). Data are expressed as means ± SD or median (interquartile ranges). The Kolmogorov-Smirnov test was used to examine the distribution of variables. Comparisons of baseline data among the groups were performed using One-way ANOVA followed by Tukey’s test or Kruskal Wallis test followed by Dunn’s Multiple Comparison Test. Spearman or Pearson correlation coefficients were calculated to describe crude associations between variables (bivariate correlation) and the effect of potential confounding factors was tested in multivariate linear regression models. A *p*-value of < 0.05 was considered statistically significant.

## RESULTS

The baseline characteristics of the study participants are described in Table 1. As expected, systolic blood pressure (SBP) and diastolic blood pressure (DBP), weight, body mass index (BMI), neck, hip and waist circumferences, body fat percentage, and body fat mass were higher in obese when compared with normal-weight subjects (*p *< 0.0001).

Levels of salivary FRAP and SH groups were significantly elevated in the obese group when compared with the normal-weight group (*p *< 0.05 and *p *< 0.0001, respectively). There were no significant differences in levels of salivary uric acid among the groups. The degree of lipid peroxidation, measured by levels of salivary TBARS, were significantly higher in the obese group compared with the normal-weight group (*p *< 0.0001), as shown in Table 2.

**Table 2:** Concentration of salivary antioxidants and lipid peroxidation in the groups studied

		Groups	

	Normal weight	Overweight	Obese
N	54	27	68
Uric acid (mg/dL)	1.7 (1.4 – 2.4)	1.8 (1.4 – 2.6)	1.9 (1.4 – 2.3)
FRAP (mmol/L)	0.37 (0.29 – 0.46)	0.38 (0.35 – 0.48)	0.45 (0.35 – 0.59^)a^
SH groups (nmol P-SH/mL)	15.1 ± 8.9	20.9 ± 8.7^b^	22.22 ± 8.8^b^
TBARS (mmol/L)	20.7 ± 7.2	23.4 ± 7.9	28.2 ± 11.6^b^

Data are expressed as means ± SD or median (interquartile ranges). Normality was assessed by Kolmogorov-Smirnov test. Data were processed for analysis, where One-way ANOVA followed by Tukey’s test, and Kruskal-Wallis test followed by Dunn’s Multiple Comparison Test.

^a^*p* < 0.05, ^b^*p* < 0.0001 compared to normal weight group.

Serum levels of FRAP, uric acid, and TBARS were significantly elevated in the obese group when compared with the normal-weight group (*p *< 0.05, *p *< 0.0001, and *p *< 0.05, respectively). In contrast, levels of SH groups were significantly decreased in the obese group when compared with the normal-weight group (*p *< 0.0001), as shown in Table 3.

**Table 3:** Concentration of serum antioxidants and lipid peroxidation in the groups studied

		Groups	

	Normal weight	Overweight	Obese
N	54	27	68
Uric acid (mg/dL)	3.8 ± 1.0	4.2 ± 1.0	4.7 ± 1.7^b^
FRAP (mmol/L)	0.89 ± 0.20	0.98 ± 0.25	1.05 ± 0.22a
SH groups (nmol P-SH/mL)	143.6 ± 21.7	123.2 ± 25.1	105.4 ± 24.2^bc^
TBARS (mmol/L)	5.1 (4.6 – 6.5)	6.0 (5.0 – 7.4)	6.6 (5.4 – 8.3)a

Data are expressed as means ± SD or median (interquartile ranges). Normality was assessed by Kolmogorov-Smirnov test. Data were processed for analysis, where One-way ANOVA followed by Tukey’s test, and Kruskal-Wallis test followed by Dunn’s Multiple Comparison Test.

^a^* p* < 0.05, ^b^*p* < 0.0001 compared to Normal Weight Group, ^c^*p* < 0.0001 compared to Overweight Group.

Correlation analyses were performed between antioxidant parameters and salivary and serum lipid peroxidation levels among the study groups. As shown in Table 4, there were significant correlations between salivary and serum levels of uric acid (*r *= 0.5162, *p *< 0.0001) and FRAP (*r *= 0.4361,* p *= 0.001) in the normal-weight group. This positive correlation was also observed in the obese group (*r *= 0.4205 and *p *= 0.0004 for uric acid, and *r *= 0.4482 and *p *= 0.0001 for FRAP), as shown in Table 5. No other positive correlations were observed in the other groups and parameters.

**Table 4:** Correlation between antioxidants salivary and serum in normal weight group

	Saliva	Serum	*p* and *r* value
Uric Acid (mg/dL)	1.7 (1.4 – 2.4)	3.8 ± 1.0	*r* = 0.5162 *p* < 0.0001
FRAP (mmol/L)	0.37 (0.29 – 0.46)	0.89 ± 0.20	*r* = 0.4361 *p* = 0.001

Data are expressed as means ± SD or median (interquartile ranges). Normality was assessed by Kolmogorov-Smirnov test. Data were processed for analysis by Pearson or Spearman correlation.

**Table 5:** Correlation between antioxidants salivary and serum in group obese

	Saliva	Serum	*p* and *r* value
Uric Acid (mg/dL)	1.9 (1.4 – 2.3)	4.7 ± 1.7	*r* = 0.4205 *p* = 0.0004
FRAP (mmol/L)	0.45 (0.35 – 0.59)	1.05 ± 0.22	*r *= 0.4482 *p* = 0.0001

Data are expressed as means ± SD or median (interquartile ranges). Normality was assessed by Kolmogorov-Smirnov test. Data were processed for analysis by Pearson or Spearman correlation.

## DISCUSSION

Sampling of human saliva is an attractive means to diagnose and monitor diseases since the saliva may be collected easily and noninvasively. Saliva has been widely used to study a variety of molecules and biochemical substances. Salivary analysis may also be helpful in predicting the development of future diseases (26,27). In the present study, we investigated antioxidant parameters and markers of lipoperoxidation in the saliva of healthy young adults with obesity and overweight to verify if the excessive body fat would influence these parameters. We then correlated the results in the saliva with those in the serum.

The nonenzymatic parameters evaluated in this study were selected based on their specific antioxidant properties (28): (i) the FRAP test quantifies the general capacity of the saliva to chelate and inactivate metal ions (mainly Fe^2+/3+^) involved in the formation of highly reactive ROS/RNS, such as hydroxyl radicals (using the Fenton reaction); (ii) uric acid is both a preventive (chelating) antioxidant and a scavenger of free radicals that have already been produced; and (iii) SH groups are considered the nonenzymatic antioxidant frontline in most body fluids, and their measurement accurately identifies the redox (pro/antioxidant) balance in the saliva.

TBARS are products of lipid peroxidation often used to determine the balance between oxidation and antioxidation (29). The present data demonstrated an increase in TBARS concentration (*p *= 0.0002) in the saliva and serum (*p *< 0.05) in obese patients compared with non-obese individuals. These results suggest an increased generation of reactive species with potential to cause damage to cell membranes. Physiologically, an increase in O_2_ and H_2_O_2 _production can increase the levels of TBARS. Obesity associated with a dyslipidemic profile, characterized by high levels of triglycerides and low-density lipoprotein (LDL) and low levels of high-density lipoprotein (HDL), promotes lipid peroxidation through formation of free radicals and ROS. Lipid oxidation generates some products, including TBARS, F_2_-isoprostanes, and MDA, which are used as markers of oxidative stress (30).

SH groups (mixed disulfides of proteins and low-molecular-weight thiols) are very early products of protein oxidation generated during oxidative stress, formed a few seconds after oxygen radicals are generated. As a result, the assessment of the extent and specificity of this process during oxidative stress is one of the best measurements of the primary effects of oxygen radicals. S-thiolation has been correlated in many cases with changes in protein function. Hence, the estimation of salivary levels of protein thiol indicates the status of the oxidative stress (31,32). Many studies have observed changes in protein thiols, including alterations in glutathione levels in various diseases in which oxidative stress occurs (33,34). Oxidative stress is considered to be involved in the pathogenesis of insulin resistance associated with obesity (35).

In this study, salivary protein thiols were significantly increased (*p *< 0.0001) in obese patients when compared with normal-weight individuals. Similar results have been reported in studies measuring salivary and serum levels of antioxidants (peroxidase, superoxide dismutase, salivary total antioxidant status) in patients with type 1 diabetes mellitus. Increased antioxidant levels have been observed in these patients, indicating a compensation of the antioxidant systems against oxidative stress (36).

Corroborating with the above, FRAP levels were significantly higher (*p *< 0.05) in the saliva of patients in the obese group. This may reflect a compensation of the obese body against oxidative stress to neutralize reactive species since several paths generating oxidative stress are activated in obesity (37). Studies in obese diabetic subjects, compared with normal men and women matched for weight, have shown higher oxidative stress in obese individuals (12), as evidenced in this study by the increased production of TBARS.

In the acute phase of obesity, levels of antioxidants appear to be increased, but as the obesity becomes chronic, the antioxidant reserve depletes over time (9). In addition to a deficiency in antioxidants in individuals with obesity and excessive fat tissue, other mechanisms may contribute to the increased oxidative stress: hyperglycemia, increased muscle activity to bear the excessive weight, hypertension, chronic inflammation, endothelial production of EROS, and hyperleptinemia (37).

We observed no significant differences in uric acid levels in the saliva among the studied groups, although this compound has been noted to increase with increases in BMI. However, we observed that the serum of patients in the obese group had a greater concentration of uric acid. Uric acid, one of the largest hydrophilic antioxidants in the body, inhibits the action of free radicals on organic molecules, such as those that make up the cell membrane and the genetic material (26). However, the sharp increase in the concentration of uric acid appears to be a protective factor against oxidative stress, whereas its chronic increase is associated with a risk of chronic diseases (38).

Data from a series of studies have shown a strong and independent correlation between serum uric acid and insulin resistance in subjects with metabolic syndrome. Evidence has also shown that serum uric acid is a strong predictor of future development of diabetes (39,40). In this context, an important result of the present study was to detect higher concentrations of uric acid in obese individuals.

Oxidative stress occurring in obesity contributes to the oxidation of proteins in the plasma; these proteins may then undergo functional changes, in particular, loss of metabolic, enzymatic, and immunological properties (8). Protein thiol groups are the most susceptible to oxidation. Among proteins with antioxidant properties, powerful nonenzymatic antioxidants stand out such as glutathione, which has an important antioxidant action (7). Plasma thiol groups serve as antioxidants capable of removing oxidants responsible for initiating peroxidation, the main responsible for oxidative damage to proteins (40).

Our results demonstrate a significant plasma reduction in SH groups in the obese group compared with the normal-weight group. Since the serum levels of TBARS were significantly higher in the obese group, this effect was probably due to a requirement for neutralization of ROS, which are produced in greater amounts in this group. Plasma SH groups are antioxidants that scavenge oxidants that initiate peroxidation and are quantitatively the major manifestation of oxidative protein damage (18). In obesity, the increase in carbonyl protein and a concomitant reduction in plasma thiol groups may be a possible mechanism contributing to atherosclerosis, insulin resistance, and hypertension (41). These data demonstrate a consumption of thiol groups to combat overproduction of free radicals and ROS produced during oxidative stress.

The results showed a progressive increase in serum FRAP levels in relation to BMI, in addition to significantly higher levels in obese volunteers. This reinforces our line of thought in which we consider this compound to be produced in greater amounts to offset the increased oxidative stress in the obese group since FRAP levels indicate the total plasma antioxidant capacity.

The second objective of this study was to correlate the salivary and serum parameters. We found a significant positive correlation between salivary and serum levels of uric acid and FRAP in the obese and normal-weight group, suggesting that measurement of salivary antioxidants could be used in the future to assess the antioxidant status of obese patients.

In conclusion, the results of this study showed that obese young patients presented significant changes in salivary biomarkers when compared normal-weight volunteers. Changes in salivary and serum levels of FRAP, SH groups, uric acid, and TBARS in obese subjects suggest major changes in oxidative status in these individuals. These findings are particularly important since they show an oxidative and inflammatory imbalance frequently observed in obesity. These results could help determine the pathways involved in obesity-related oxidative stress that are relevant to the development of obesity in young individuals. The results demonstrated a good correlation between salivary and serum biomarkers. There was a good replication in the results and maybe in the future, salivary can be used instead of blood to measure these oxidative biomarkers. Hence, we conclude that in addition to physical characteristics, levels of salivary FRAP and SH groups are indicators of oxidative stress in young obese individuals. Salivary analysis may prove to be a noninvasive, patient-friendly technique to assess the antioxidant status in these cases.
